# Critical cardiovascular complications during non-immunosuppressive therapy for VEXAS syndrome

**DOI:** 10.1093/rap/rkaf028

**Published:** 2025-03-10

**Authors:** Yuna Saito, Satoshi Suzuki, Keigo Ikeda, Shinji Morimoto

**Affiliations:** Department of Internal Medicine and Rheumatology, Juntendo University Urayasu Hospital, Urayasu, Japan; Department of Internal Medicine and Rheumatology, Juntendo University Urayasu Hospital, Urayasu, Japan; Department of Internal Medicine and Rheumatology, Juntendo University Urayasu Hospital, Urayasu, Japan; Department of Internal Medicine and Rheumatology, Juntendo University Urayasu Hospital, Urayasu, Japan

Key message

- Mild VEXAS syndrome, if left untreated with systemic therapy, may lead to delayed critical complications.

Dear Editor, An 85-year-old male with a history of diabetes mellitus, hypertension and dyslipidaemia presented with erythema, pruritus and subcutaneous nodules on his limbs and trunk a year before his hospital visit (Fig. 1A–C). He was initially diagnosed with panniculitis after a skin biopsy and treated with glucocorticoid (GC) ointments and oral antihistamines, which provided temporary relief. However, 1 month later, painful erythematous swelling developed in the left ear (Fig. 1D). Despite antibiotic therapy initiated by a dermatologist, the swelling persisted, and 2 weeks later, the right ear became affected. Suspecting relapsing polychondritis (RP), the patient was referred to our department.

**Figure 1.**
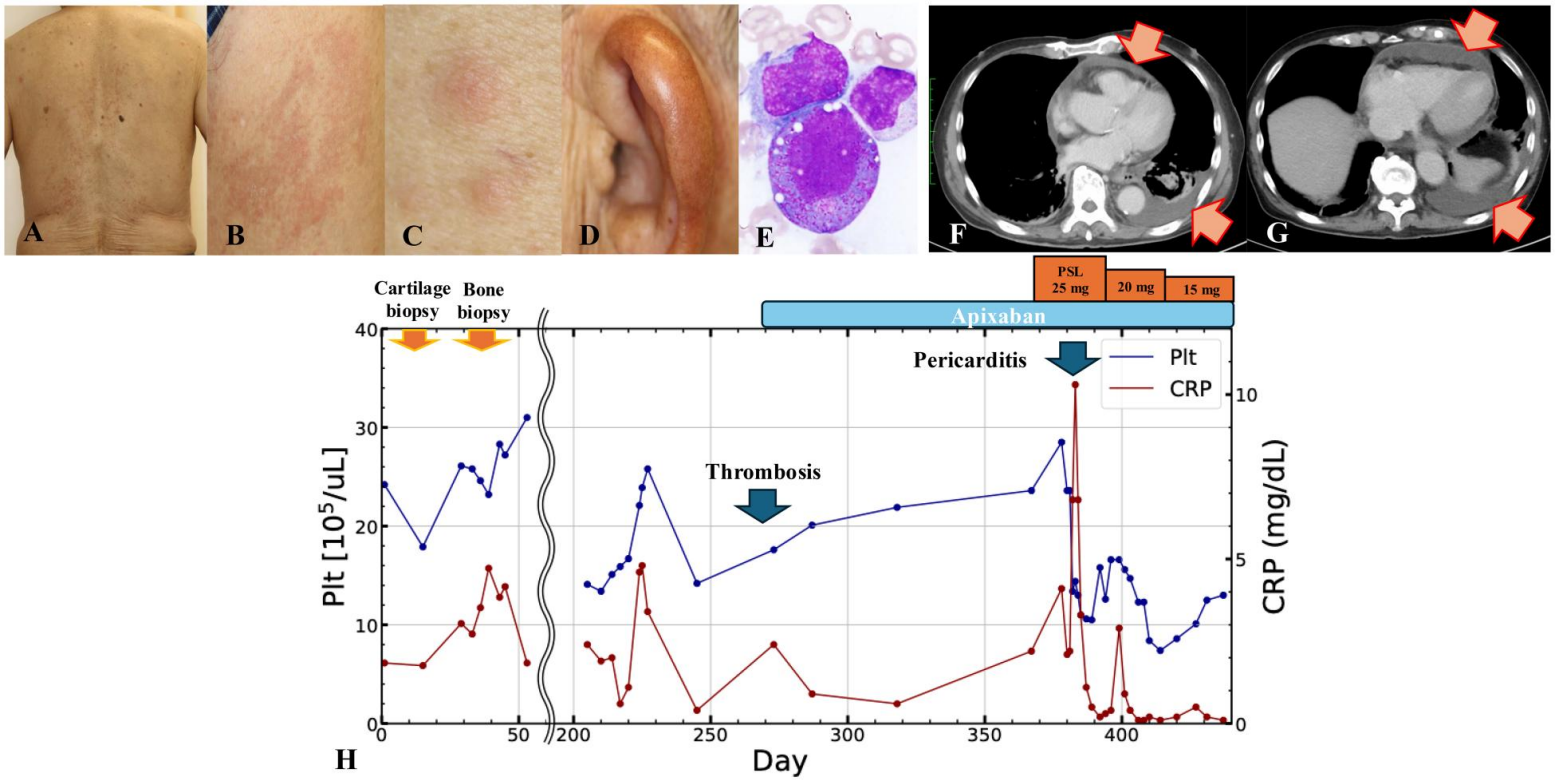
Presentation and disease course. (A–C) Erythema with pruritus evident on the limbs and trunk, along with subcutaneous nodules. (D) Left ear swelling. (E) Bone marrow biopsy: dysplasia and characteristic findings of granulocyte and erythroblast ‘vacuoles’ (magnification: ×40). (F, G) Contrast-enhanced CT scan: new pericardial and bilateral pleural effusions suggest the development of acute pericarditis and pleuritis. The arrows indicate areas of pericardial and pleural effusion. (H) Record of treatment progress for ∼400 days from initial visit to discharge from the hospital. PSL: prednisolone; PLT: platelet count

Laboratory tests showed mild elevation in inflammatory markers and macrocytic anaemia, but other blood cell counts were normal. Autoimmune disease tests were negative. Upon physical examination, the rash and ear swelling had resolved. A biopsy of the ear cartilage showed mild inflammatory infiltration and cartilage degeneration, confirming RP [1]. However, the presence of panniculitis and macrocytic anaemia raised concerns about myelodysplastic syndrome (MDS). A bone marrow biopsy revealed dysplasia with characteristic granulocyte and erythroblast vacuoles, suggesting MDS (Fig. 1E). Genetic testing identified a mutation in UBA1 (c.121A>C, p. Met41Leu), confirming VEXAS syndrome [2–5].

Systemic GC therapy was deferred as both the auricular chondritis and skin rash resolved spontaneously without airway involvement. Six months later, the patient presented with swelling in the right lower leg, and venous ultrasonography confirmed extensive deep vein thrombosis (DVT). A computed tomography (CT) scan revealed a pulmonary thromboembolism, and anticoagulation therapy with apixaban was initiated, showing subsequent improvement.

Six months later, the patient presented with dizziness and severe anaemia (haemoglobin 6.8 g/dl), receiving a blood transfusion. A contrast-enhanced CT scan showed no active bleeding sources, and gastroscopy was unremarkable. On day 3 of hospitalization, the patient developed a fever, and 8 h later, his systolic blood pressure dropped to 70 mmHg. An electrocardiogram (ECG) showed ST elevation in leads V5 and V6, and echocardiography revealed a new pericardial effusion. CT confirmed new pericardial and pleural effusions, suggesting acute pericarditis and pleuritis (Fig. 1F and G).

Differential diagnoses included sepsis, gastrointestinal bleeding and exacerbation of VEXAS syndrome. Tazobactam/piperacillin was administered because of concerns of sepsis, but all infection tests were negative. Given the possible exacerbation of VEXAS syndrome, prednisolone 25 mg/day was started (Fig. 1H), with rapid improvement in blood pressure, ECG and echocardiogram. Prednisolone was tapered, and by day 56, the dose was reduced to 15 mg/day without further hypotension. The patient was transferred to a chronic care hospital for rehabilitation before returning to our facility.

This case describes a patient with VEXAS syndrome and RP who was initially treated conservatively with topical therapy. Despite initial improvement, the patient later developed acute pericarditis and pleuritis, a rare complication of VEXAS syndrome, with an incidence of only 4.3% [6, 7]. No other cases of pericarditis in VEXAS syndrome were identified, which could be attributed to either early immunosuppressive therapy or patient mortality before such complications developed.

Two points of potential systemic GC initiation are considered. First, at the time of diagnosis, when cartilage inflammation had already improved with topical therapy, systemic GCs were not used owing to the patient's poorly controlled diabetes mellitus, which might have worsened with GC use. The second potential point was after the development of thrombosis. VEXAS syndrome has been associated with an increased risk of DVT [7], as well as myocarditis, pericarditis and pericardial effusion [7], which could justify the initiation of systemic therapy. However, given that GCs increase the risk of thrombosis, their use during the acute phase remained uncertain.

This case highlights that early systemic GC therapy for VEXAS syndrome, regardless of severity, may improve prognosis. However, prolonged GC use may lead to adverse effects, necessitating the exploration of alternative immunosuppressants to avoid such complications.

## Data Availability

The data underlying this article will be shared on reasonable request to the corresponding author.
